# Comparative metagenomic analysis of 16S RNA amplicon sequencing of bacterial population of an industrial site contaminated with halogenated anilines

**DOI:** 10.1128/mra.00856-24

**Published:** 2025-02-25

**Authors:** Olukemi Ajibola Tobun, Sunday Adekunle Adebusoye, Matthew Olusoji Ilori

**Affiliations:** 1 Department of Microbiology, University of Lagos, Akoka, Lagos, Nigeria; Montana State University, Bozeman, Montana, USA

**Keywords:** metagenomes, halogenated aniline compounds, enrichment culture, contaminated sites

## Abstract

Here, we use 16S rRNA gene sequencing to identify bacterial communities in three soil samples from contaminated soils with halogenated anilines and empty soil (serving as control) in Lagos state, Nigeria. BNE, BLB, and BLC had unique operational taxonomic units (OTUs) of 160, 1,797, and 4,815, respectively. Proteobacteria were the dominant phyla across all samples.

## ANNOUNCEMENT

Halogenated anilines (HAs) are an important group of industrial chemicals, which are predominantly used as chemical intermediates for the production of azo dye, photographic chemicals, pesticides, herbicides, pigments, plastics, solvent, and drug intermediates (1–5). The products of these chemicals are generally used in different household and agricultural applications (6). There is widespread usage, accumulation, and increasing rate of industrialization of these products for human sustainability in Nigeria, but their usage contributes high risk to human health and the environment (7–9). Industrial sites are located in Lagos State, Nigeria, and the sites are polluted with halogenated anilines. Here, we used culture-independent techniques to assess the microbial diversity of industrially polluted soils. We developed a cultivation experiment using an enrichment culture technique to obtain metagenome associated with the degradation of halogenated aniline compounds.

Genomic DNA was isolated from 1.0 g (approx.) each of soil sample from the industrial site (ISS) (Latitude 6.597038′, Longitude 3.96932′), its culture after the fourth enrichment transfer (BNE), and an empty land that serves as a control soil sample (BLC) (6.646945′, Longitude 3.506213′). It was carried out with the Fast DNA Spin Kit for Soil (MP Biomedicals) using FastPrep Cell Disruptor FP120 (Qbiogene, Heidelberg, Germany) at 6.5 speed for 30 s, according to the manufacturer’s instruction. Feasible interference of humic substances in the extracted DNA was removed by addition of skim milk (20 mg per 500 of sample) to the sample in lysis matrix as recommended by Takada and Matsumoto (10). DNA was purified and visualized in an ethidium bromide stained 1% (w/v) agarose gel using UV trans-illumination, while quantification was via UV–Vis photo-spectrometry using the Epoch Spectrometer system (BioTek, Winooski, VT, USA).

Libraries were constructed at ChunLab Inc. using the Illumina MiSeq platform, where the quality of the constructed libraries was checked with the Agilent 2100 Bioanalyzer System (Agilent Technologies, Palo Alto, CA, USA) using a DNA 7500 chip at ChunLab Inc. (Seoul, South Korea) and thereafter quantified using the Quanti-iT PicoGreen dsDNA Assay kit (Invitrogen) according to the manufacturer’s instructions. The bacterial culture pellet was suspended in PBS buffer and then centrifuged in a ZR Bashing Beads lysis tube. The supernatant was passed to a Zymo-Spin filter, filtered, and diluted with genomic lysis buffer before centrifugation again. DNA pre-wash buffer was added, then the Zymo-Spin II CR was washed with g-DNA and wash buffer. The 1.5 mL Zymo-Spin clean solution was then transferred and mixed with DNA elution buffer. Following centrifugation, the ultra-pure DNA was broken with enzymes, and size selection was performed.

A short DNA fragment was removed using CleanPCR (CleanNA, Netherlands), and sequencing was performed using Illumina, MiSeq Reagent Kit v2 (500 cycles) of Illumina MiSeq platform at ChunLab Inc., Seoul National University, Seoul, Korea. Metagenome raw reads were processed beginning with quality check and filtering of low-quality (<Q25) reads using Trimmomatic 0.32 software (11). The paired-end sequence of the same strand of PCR amplicon was merged based on overlapping sequence information using PANDAseq software (12). Non-specific amplicons were identified and removed using the HMMER program-based search to exclude singleton sequences (13), while sequence denoising was performed with DUDE-Seq software (14). Sequences were de-replicated, and non-redundant reads were extracted via UCLUST-clustering (15). UCHIME (16) was used for detection and removal of chimera, while the remaining non-chimeric sequences were clustered into operational taxonomic units (OTUs) using UCLUST (15). Query sequences that were matched with the reference sequences in the EzBioCloud database (https://www.ezbiocloud.net/) by ≥97% similarity were considered to be at the species level, while <97% similarity cut-offs were used for genus or higher taxonomic levels. Default parameters were used except where otherwise noted. Table 1 shows the 16S RNA amplicon sequencing statistics. The target reads for BNE, BLB, and BLC samples were 65,259, 61,554, and 61,763, respectively. OTUs in all samples, with the least diverse sample possessing 160 and the most diverse containing 4,983.93 OTUs.

**TABLE 1:** 16S RNA amplicon sequencing statistics collected from industrial sites^*a*^

Attribute	Value		
	BNE	BLB	BLC
Target reads	65,259	61554	61,763
OTUs	160	1,797	4,815
ACE	186.75	1907.28	4,983.93
CHAO	175.49	1,858.45	4,884.21
Jackknife	197	2,008	5,215
NP Shanon	1.13	4.98	7.29
Shanon	1.12	4.94	7.29
Simpson	0.46	0.04	0
Phylogenetic diversity	380	2,467	4,967
Good coverage	99.94	99.66	99.35

^*a*^ BLB: Industrial soil sample, BNE: Enrichment culture (last transfer), and BLC: Control soil sample.

The Shannon index (SI) assigned BLB and BNE diversity values of 4.94 and 1.12, respectively. In contrast to BLB and BNE, BLC showed the largest diversity (7.29).

**Fig 1:**
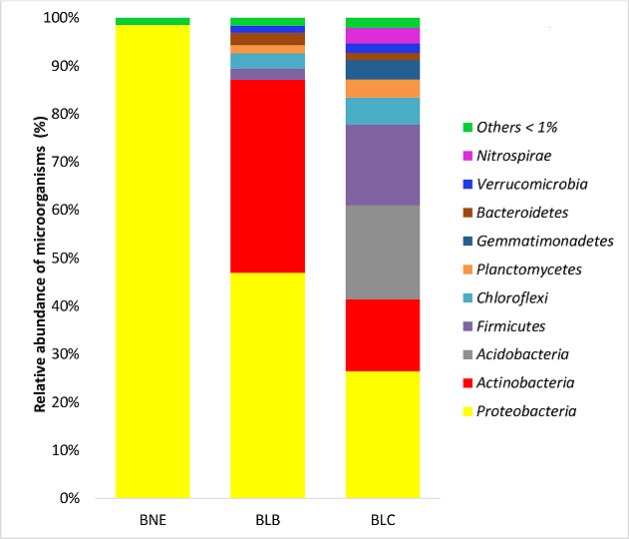
Relative abundance of major phyla (≥ 0.05%).

Figure 1 depicts the bacterial taxonomic profile of three ISS microbiomes at the phylum level. Figure 1 shows that proteobacteria dominated BNE, BLB, and BLC at 98.52%, 46.93%, and 26.46%, respectively.

## Data Availability

Raw sequences generated in the present study have been deposited with NCBI Genbank under the following SRA accession numbers; SRR31046205, SRR31046206, and SRR31046204, for enrichment culture (BNE), industrial soil sample (BLB) and control soil sample (BLC), respectively (https://www.ncbi.nlm.nih.gov/sra/SRR31046204-6). Also, metagenome sequences obtained have been deposited in GenBank under the bioproject and biosample numbers PRJNA1140845 and SAMN42862938, respectively, (https://www.ncbi.nlm.nih.gov/nuccore/?term=PRJNA1140845; https://www.ncbi.nlm.nih.gov/nuccore/?term=SAMN42862938%2C).
